# Characterization of flavor volatile compounds in industrial stir‐frying mutton sao zi by GC‐MS, E‐nose, and physicochemical analysis

**DOI:** 10.1002/fsn3.2019

**Published:** 2020-12-10

**Authors:** Shuang Bai, Yongrui Wang, Ruiming Luo, Dan Ding, He Bai, Fei Shen

**Affiliations:** ^1^ School of Agriculture Ningxia University Yinchuan China

**Keywords:** E‐nose, GC‐MS, Mutton sao zi, stir‐frying, volatile compounds

## Abstract

This study aims to investigate the flavor changes of industrial stir‐frying mutton sao zi, a mutton product popular in the northwest of China, at different stir‐frying stages. Electronic nose (E‐nose) was used to recognize mutton sao zi odors at different processing time points, and the individual volatile compounds were further identified by the solid‐phase microextraction (SPME) combined with gas chromatography–mass spectrometry (GC‐MS). A total of 105 volatile compounds were detected by GC‐MS, of which 51 were major volatile compounds. Additionally, GC‐MS and E‐nose data of the samples were also correlated with the fatty acids, crude composition (moisture, fat, protein), and amino acids. The stir‐frying time and temperature may be the critical contributors to different flavors of industrial stir‐frying mutton sao zi. The signal intensities of W1S, W1W, W2S, W2W, and W3S sensors positively correlate with protein, fat, and 18 amino acids, but negatively with SFA and moisture. Hence, this study explored the flavor changes of industrial stir‐frying mutton sao zi by E‐nose and SPME‐GC‐MS for the first time, providing an insight into the industrial production and flavor control stir‐frying machine of stir‐frying mutton products with household flavor.

## Graphical abstract

The correlation between E‐nose, GC‐MS, and crude composition, 18 amino acid,and fatty acids indicates that high contents will contribute to E‐nose to discriminate different processing stages of mutton sao zi products under Chinese‐style stir‐frying conditions.

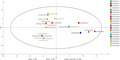

## 1 INTRODUCTION

Mutton sao zi is a famous stir‐fried meat product in northwest China, and its main ingredient is the Tan sheep meat. The mutton sao zi is traditionally produced by stir‐frying which cooks the meat with a unique culinary quality compared with other frying methods like deep‐frying and pan‐frying. The industrial stir‐frying processing of mutton sao zi is commonly in a large wok, while the household processing in a shallow vessel (a Chines wok). This stir‐frying approach has shown distinct advantages for meat processing over other cooking methods. For example, stir‐frying offers a superior quality and flavor and a better retention of trace elements as vitamin B_6_, vitamin B_1_, iron, magnesium, and zinc, when compared to other cooking methods like microwave cooking and roasting (Adler‐Nissen, 2002). There are various mutton products with different taste and flavor in different countries, such as fermented mutton sausage(Jia et al., 2020; Zhao et al., 2011) and mutton shashlik (Sun, et al., 2010) in China, mutton patties (Pathera et al., 2016) and dried mutton (Jayathilakan et al., 2012) in India and lamb spine (Karakal et al., 2013) in Turkey. But there are no stir‐fried mutton products. Mutton sao zi manufactured by traditional stir‐frying technologies is particularly popular among northwest Chinese for its unique characteristic flavors and rich nutrition.

E‐nose and GC‐MS are two alternative approaches to determine the odors. E‐nose makes significant contributions to determination of flavors, offering a comprehensive and fast alternative to assess meat quality (Zhang et al., 2019). GC–MS is one of the main methods to identify traditional meat volatile compounds, such as sauce spareribs (Shi et al., 2020), cold‐smoked Spanish mackerel (Huang et al., 2019), beef (Aaslyng & Meinert, 2017), and so on. Solid‐phase microextraction (SPME) is widely applied to collect volatiles from meat, as it is environment‐friendly, fast, and simple operation (Dominguez et al., 2019). Although they used GC‐MS and E‐nose to study the composition and correlation of the volatile compounds, they ignored the role of amino acid, fatty acid, or crude composition in meat products at different processing stages. In another word, the flavor changes of the mutton during the stir‐frying process have been less explored.

Hence, in this study, a systematic analysis on the changes of volatile compounds at different operating stages of traditional industrial stir‐frying mutton sao zi was performed. The extracted and identified volatile components from combination of SPME and GC‐MS might contribute to evaluate the flavor formation at different processing time points of stir‐frying mutton sao zi. The associations between content of volatile compounds and the intensities of E‐nose sensors, amino acid, crude composition, and fatty acids were elucidated. This work may provide a reference for controlling the mutton sao zi of stir‐frying production chain and a technical guidance for industrial production, to improve the flavor quality.

## 2 MATERIALS AND METHODS

### 2.1 Chemicals

2‐Methyl‐3‐heptanone (99.50%) and n‐alkanes (C7‐C30) of chromatographic grade were bought from Dr. Ehrenstorfer GmbH and Sigma. The 18 amino acids mixed standard were bought from sigma.

### 2.2 Preparation of samples

The mutton sao zi samples was taken from a commercial meat company in Yinchuan (Ningxia, China) and prepared according to the traditional method of Hui people in Ningxia, China. Firstly, 130kg of fresh Tan sheep hind leg meat with a fat‐lean ratio of 3:7 was cleaned and dice into 1 cm × 1 cm × 1 cm (length × width ×thickness) cubes. Secondly, cooking wine and soy sauce were added to marinate the meat for 10 min. Then, a small amount of sesame oil was heated in the pot to 80°C and poured into the marinated mutton. Finally, the meat was stir‐fried for 35 min according to the stir‐frying process. The stirring rate of stir‐frying mutton sao zi was 30 times/min. Samples at predetermined time points (0, 5, 10, 15, 20, 25, 30, and 35 min) were stored at −20°C for following experimental analysis.

### 2.3 Determination of crude composition, fatty acid and free amino acids

Moisture, protein, and fat were determined as described by Association of Official Agricultural Chemists (AOAC, 2012). The moisture content was calculated by a percentage of the weight loss of the samples before and after drying, the crude protein content quantified by the automatic Kjeldahl analyzer (KDN‐520, Lvbo) and the fat content extracted by the Soxhlet method which calculates the percentage of weight loss before and after extraction.

The fatty acids compositions were determined by GC analysis with a slight adjustment to the method described (Shi et al., 2020). Briefly, fat was saponified in a water bath (HWS‐12) at 70°C for 1 hr and then methylated by reacting with 7ml 15% boron trifluoride methanol. The saturated NaCl solution and n‐hexane (C11) were added and then stratified. The gas chromatography (Shimadzu GC‐2010 plus) was equipped with a flame ionization detector. The upper solution was injected into a GC and separated on a SP‐2560 column (100 m × 0.25 mm × 0.25 mm, Supelco). The fatty acid was identified by comparison with standard certificate (37 types of fatty acids, Sigma‐Aldrich). Results were expressed as a percentage of its peak area to the total peak area. The compounds were identified by comparing and matching their mass spectra of each component with NIST 14 mass spectra database.

The analysis of 16 amino acids was performed with LA8080 amino acid analyzer (Hitachi, Japan). 200 mg of the sample was homogenized by sterile homogenizer (HX‐11L) and put in a hydrolytic tube. 1 0ml hydrochloric acid of 6 mol/L (0.5% mercaptoacetic acid) was added to the hydrolytic tube and frozen in a refrigerant for 3 min. The process to vacuum (close to 0 Pa) and fill with high purity nitrogen was repeated for 3 times. Then, the sealed hydrolytic tube was placed in drying oven of a constant temperature (110 ± 2) °C with nitrogen filled or screw cap tightened. Following the hydrolysis for 22 hr, it was taken out for cooling. After the filtrate was filtered, the hydrolytic tube was washed with deionized water for several times. The vacuum dryer was dried at 40 ~ 50 degrees, and the residue was dissolved by 0.02moL/L hydrochloric acid to dilute the volume. After filtration, the solution was analyzed by a 0.45 μm filtration membrane.

The content of cystine was slightly different from that of 16 amino acids by LA8080 amino acid analyzer. After vacuum drying and filtering, the cystine was added with 3ml sodium citrate buffer solution to a constant volume.

The analysis of tryptophan was performed with LC‐20 A high‐performance liquid chromatography (SHIMADZU). 100 mg of the sample was homogenized by sterile homogenizer (HX‐11L, China) and put in a polytetrafluoroethylene tube. 1.5 ml of 4mol/L lithium hydroxide solution was added, filled with nitrogen for 1 min, sealed and put into a drying oven at a constant temperature of 110 ± 2°C for hydrolysis for 20 hr. After cooling to room temperature, the hydrolysate was quantitatively transferred to a 25ml volumetric flask with 0.0085 mol/L sodium acetate buffer solution for constant volume. The 0.45 μm filtration membrane was used for analysis.

### 2.4 Electronic nose detection

The PEN 3.5 electronic nose (Airsense) is an array of chemical gas sensors for measurement of volatile compounds within the headspace over stir‐frying mutton sao zi samples. The sensor array is composed of 10 gas sensors with metal oxide semiconductors of different selectivity and sensitivity to volatile compounds, with certain specificity. The sensors include W1C (aromatic compounds), W1S (methane, broad range of compounds), W1W (sulfur compounds, terpenes), W2S (broad range, alcohols), W2W (aromatics and organic sulfur compounds), W3C (ammonia, aromatic compounds), W3S (methane and aliphatic compounds), W5C (alkanes and aromatics), W5S (nitrogen oxides), and W6S (hydrocarbons). Before the analysis, 5 g of minced meat samples was put into 20 ml airtight vials and incubated in the water bath at 25°C for 20 min. The chamber was flushed with clean air until the sensor signal returned to the baseline before testing new samples.

### 2.5 Volatile compounds analysis

A head space solid‐phase microextraction (HS‐SPME) fiber assembly combined with a GC‐MS system (GC‐MS 2010 plus, SHIMADZU) was used for detecting volatile compounds from the selected samples. A 3 g minced meat sample and 3 ml saturated sodium chloride solution were added into a 20 ml head space vial (Supelco) and homogenized with a glass rod for 2 min, and then sealed with a silicone septum. The vials and their contents were pre‐heated at 60°C for 20 min to the insertion of the SPME fiber (DVB/CAR/PDMS‐50/35 μm, Supelco) into the headspace where it was held for 30 min. The volatile compounds were separated using DB‐WAX polar analytical column (30 m × 0.25 mm × 0.25 μm, Agilent) in GC‐MS system (GC‐MS 2010 plus, SHIMADZU).

The adsorbed SPME fiber was inserted into the injection port of GC‐MS after extraction, and desorbed at 250°C for 5 min. Helium served as the carrier gas with a flow rate of 2.0 ml/min (constant flow). The temperature gradient in GC oven was as follows: initial 40°C for 3 min, 40°C to 200°C under a 5°C/min rate, and 200°C to 230°C at which holding for 3 min. Electron impact (EI) mode was used at 70 eV, with a full scan range from 50 to 350 amu. The interface temperature 250°C, ion source temperature 230°C, and a solvent delay time of 2.5 min were adopted. The mass spectra of volatiles detected from samples were compared and matched with the mass spectra from NIST 14.0 and the standard compound retention index (retention index, RI). Semi‐quantitative determinations were obtained by using 2‐methyl‐3‐heptanone as an internal standard. The volatile compounds acquired were identified for the reverse match factor (similarity > 700) and retention index (RI). The C7 ~ C30 n‐alkanes were employed to calculate linear RI of volatile compounds.

### 2.6 Statistical analysis

All statistical analyses are the averages of three biological replicates. Analysis of variance (ANOVA) and principal component analysis (PCA) were performed by IBM SPSS 24.0 and SIMCA 14.0. The correlation analysis was performed on origin 2020b by using correlation plot package. Origin 2020b and Microsoft Office 2019 were used to radar chart and plot.

## 3 RESULTS AND DISCUSSION

### 3.1 Chemical composition of mutton sao zi

#### 3.1.1 Crude composition of Mutton sao zi

The chemical composition (moisture, protein, and fat) characteristics of mutton sao zi were showed great variations among different processing time points as shown in Table 1. During processing of the meat, the moisture of product drops, depending largely on the processing time. With the extension of processing time of the average moisture content of chicken nuggets decreased during frying at 175°C and 190°C (Bansal et al., 2015). Moisture content ranged from 69.79% for stir‐frying 0 min samples to 47.11% for stir‐frying 35 min samples. This could be attributed to the difference in stir‐frying time. At the same temperature, longer time stir‐frying may cause greater moisture loss.

**Table 1:** Crude composition (%) of mutton sao zi from different processing time (means, ±*SD*)

	Moisture (%)	Protein (%)	Fat (%)
Stir‐frying 0 min	69.7875 ± 1.3631^a^	18.1082 ± 0.5078^e^	9.3867 ± 0.3062^f^
Stir‐frying 5 min	66.7515 ± 1.0321^b^	19.2001 ± 0.4803^e^	9.9774 ± 0.3163^f^
Stir‐frying 10 min	62.3706 ± 1.1484^c^	22.7207 ± 0.5629^d^	11.8590 ± 0.3869^e^
Stir‐frying 15 min	54.8030 ± 0.9758^e^	26.5396 ± 0.7065^a^	12.6731 ± 0.4134^d^
Stir‐frying 20 min	50.4059 ± 1.0352^de^	29.0710 ± 0.6124^c^	14.5223 ± 0.4738^b^
Stir‐frying 25 min	48.1095 ± 0.9601^d^	29.2929 ± 0.6171^c^	15.4670 ± 0.5046^a^
Stir‐frying 30 min	47.3458 ± 0.8970^f^	32.4301 ± 0.6831^b^	13.7886 ± 0.4498^c^
Stir‐frying 35min	47.1097 ± 0.8917^f^	32.2354 ± 0.6790^b^	13.7059 ± 0.4472^c^

Note

The values denote mean ± standard deviation, *n* = 3. Means within a same row with alphabets (a, b, c) are significantly different (*p* < .05).

The protein undergoes Maillard and thermal degradation reaction during heating, and further has some contributions to the quality and flavor of meat products (Wall et al., 2019). After the raw meat was stir‐fried for 35 min, the protein content increased from 18.11% to 32.24%. In our research, the protein content did not differ between 0 min and 5 min, 20 min and 25 min, and 30 min and 35 min (*p* > .05). The increase of protein content in stir‐frying 0–35 min may be due to the decrease of water content, leading to an increase in protein proportion. Throughout the cooking process, meat proteins were denatured by heat with subsequent loss of water‐holding capacity (Tornberg, 2005), but most of the loss appeared to be water, resulting in higher contents of other components in the cooked meat.

Fat is a major precursor of cooked meat flavor, and lipid oxidation is the leading reaction during cooking which helps uncooked meat with bloody taste and little aroma develop the meat flavor (Khan et al., 2015). The raw meat stir‐fried for 20 min had the fat content gradually increased (to 9.39%, 9.98%, 11.86%, 12.67%, 14.52%, and 15.47%, respectively), which may be attributable to the moisture loss during the stir‐frying process. Boiled samples contained a lower content of protein and fat than the dry heat (baked and fried) treated ones, probably due to a dilution effect of the major water retention (Tavares et al., 2018). The moisture, protein, and fat values did not show significant differences (*p* > .05).

#### 3.1.2 Fatty acids profiling of Mutton sao zi

Fatty acids in industrially produced mutton sao zi showed significant difference in content at different processing stages as shown in Table 2. Fatty acids are involved in various “technological” aspects of meat quality. The oxidized fatty acids contributed to increase the flavor intensity of meat through lipid oxidation products, this propensity to oxidize is important in flavor development during cooking (Wood et al., 2004). The content of saturated fatty acid (SFA) in the samples timed 0, 5, 10, 15, 20, 25, 30, and 35 min was 42.10%, 40.99%, 40.94%, 36.27%, 35.345%, 37.51%, 32.07%, and 32.02% respectively.

**Table 2:** Fatty acids profile (g/100g of total fatty acids) in mutton sao zi from different processing time (means, ±*SD*)

Fatty acids	Stir‐frying 0min	Stir‐frying 5min	Stir‐frying 10min	Stir‐frying 15min	Stir‐frying 20min	Stir‐frying 25min	Stir‐frying 30min	Stir‐frying 35min
C6:0	0.6116 ± 0.0272^a^	0.0994 ± 0.0036^c^	0.204 ± 0.0059^b^	0.0744 ± 0.0016^d^	0.0125 ± 0.0007^e^	0.0796 ± 0.0019^d^	0.0681 ± 0.0047^d^	0.0674 ± 0.0035^d^
C8:0	0.3254 ± 0.0144^b^	0.2358 ± 0.008^e^	0.2873 ± 0.0077^c^	0.3951 ± 0.0079^a^	0.2616 ± 0.0149^d^	0.1888 ± 0.0045^f^	0.1615 ± 0.011^g^	0.1602 ± 0.0083^g^
C11:0	0.0659 ± 0.0029^ab^	0.0687 ± 0.0023^a^	0.0645 ± 0.0018^b^	0.0241 ± 0.0022^cd^	0.0132 ± 0.0008^e^	0.0253 ± 0.0006^c^	0.0217 ± 0.0015^d^	0.0215 ± 0.0011^d^
C13:0	0.2459 ± 0.0109^a^	0.2366 ± 0.008^a^	0.1854 ± 0.005^b^	0.0766 ± 0.0011^c^	0.0798 ± 0.0045^c^	0.0317 ± 0.0008^d^	0.0271 ± 0.0019^d^	0.0272 ± 0.0014^d^
C14:0	2.9109 ± 0.076^c^	3.9921 ± 0.1352^a^	3.3058 ± 0.0946^b^	1.4409 ± 0.0305^e^	1.656 ± 0.0942^d^	1.4957 ± 0.0352^e^	1.2791 ± 0.0872^f^	1.2749 ± 0.0656^f^
C15:0	0.3781 ± 0.0168^a^	0.2962 ± 0.0107^b^	0.2515 ± 0.0067^c^	0.1974 ± 0.0026^d^	0.1588 ± 0.009^e^	0.2014 ± 0.0047^d^	0.1722 ± 0.0117^e^	0.173 ± 0.0089^e^
C16:0	19.7201 ± 0.877^a^	16.4429 ± 0.5567^de^	16.8953 ± 0.7627^cde^	17.8955 ± 0.2026^bc^	17.6665 ± 1.005^bcd^	18.7609 ± 0.4415^ab^	16.0438 ± 1.0931^e^	16.1061 ± 0.8227^e^
C17:0	1.9441 ± 0.0507^a^	1.7781 ± 0.0638^b^	1.8093 ± 0.0482^b^	1.1691 ± 0.0182^d^	1.1302 ± 0.0643^d^	1.2637 ± 0.0297^c^	1.0807 ± 0.0737^d^	1.0856 ± 0.0554^d^
C18:0	13.113 ± 0.5832^b^	14.2662 ± 0.483^a^	14.4888 ± 0.4149^a^	11.1914 ± 0.2248^c^	11.0413 ± 0.6282^c^	11.4914 ± 0.2704^c^	9.8271 ± 0.6696^d^	9.8314 ± 0.5039^d^
C20:0	0.3162 ± 0.0082^a^	0.0936 ± 0.0032^e^	0.232 ± 0.0066^b^	0.0239 ± 0.0028^g^	0.0577 ± 0.0033^f^	0.1651 ± 0.0039^c^	0.1412 ± 0.0096^d^	0.1408 ± 0.0072^d^
C21:0	1.2561 ± 0.0559^e^	1.6316 ± 0.0585^d^	2.1897 ± 0.0583^c^	2.6724 ± 0.0567^a^	2.6774 ± 0.1523^a^	2.8184 ± 0.0663^a^	2.4102 ± 0.1642^b^	2.4211 ± 0.1236^b^
C22:0	0.2501 ± 0.0111^a^	0.1136 ± 0.0039^b^	0.1174 ± 0.0034^b^	0.0876 ± 0.001^cd^	0.0468 ± 0.0027^e^	0.0949 ± 0.0022^c^	0.0812 ± 0.0055^d^	0.0806 ± 0.0042^d^
C23:0	0.3276 ± 0.0085^b^	0.894 ± 0.0321^a^	0.351 ± 0.0101^b^	0.1778 ± 0.0038^e^	0.2791 ± 0.0159^c^	0.2843 ± 0.0067^c^	0.2432 ± 0.0166^d^	0.2413 ± 0.0125^d^
C24:0	0.4992 ± 0.0222^d^	0.7104 ± 0.0255^b^	0.4613 ± 0.0123^e^	0.8013 ± 0.0085^a^	0.2517 ± 0.0143^f^	0.5862 ± 0.0138^c^	0.5014 ± 0.0342^d^	0.5036 ± 0.0257^d^
C14:1	0.3026 ± 0.0079^a^	0.2795 ± 0.01^b^	0.2334 ± 0.0063^c^	0.122 ± 0.0019^d^	0.1186 ± 0.0068^de^	0.1272 ± 0.003^d^	0.1087 ± 0.0074^e^	0.1063 ± 0.0056^e^
C15:1	1.5436 ± 0.0686^c^	3.207 ± 0.1086^a^	2.6217 ± 0.0751^b^	1.4157 ± 0.03^d^	0.6728 ± 0.0382^e^	0.2575 ± 0.006^f^	0.2202 ± 0.015^f^	0.2212 ± 0.0113^f^
C16:1	1.987 ± 0.0519^b^	4.6627 ± 0.1579^a^	4.6802 ± 0.1246^a^	1.416 ± 0.0228^c^	1.2942 ± 0.0737^cd^	1.3826 ± 0.0325^c^	1.1823 ± 0.0806^d^	1.1837 ± 0.0607^d^
C17:1	1.0597 ± 0.0471^c^	1.5995 ± 0.0574^a^	1.5296 ± 0.0438^b^	0.4565 ± 0.0097^e^	0.6069 ± 0.0346^d^	0.4167 ± 0.0098^fg^	0.3563 ± 0.0243^g^	0.3579 ± 0.0183^g^
C18:1n9t	0.0958 ± 0.0025^a^	0.0713 ± 0.0025^b^	0.0681 ± 0.0018^c^	0.0198 ± 0.0007^e^	0.0273 ± 0.0016^d^	0.0249 ± 0.0006^d^	0.0212 ± 0.0015^e^	0.0204 ± 0.0011^e^
C18:1n9c	40.0826 ± 1.0781^b^	34.5804 ± 1.1707^c^	35.6111 ± 1.0196^c^	44.6795 ± 0.947^a^	44.9982 ± 2.56^a^	46.5156 ± 1.0945^a^	39.7789 ± 2.7104^b^	39.7582 ± 2.0397^b^
C20:1	0.0967 ± 0.0043^c^	0.2311 ± 0.0083^a^	0.2087 ± 0.0056^b^	0.0951 ± 0.002^c^	0.0857 ± 0.0049^d^	0.0783 ± 0.0018^d^	0.0669 ± 0.0046^e^	0.0653 ± 0.0034^e^
C22:1n9	0.2445 ± 0.0064^a^	0.0906 ± 0.0031^b^	0.0536 ± 0.0015^d^	0.0716 ± 0.0015^c^	0.0595 ± 0.0034^d^	0.0678 ± 0.0016^c^	0.0579 ± 0.004^d^	0.0582 ± 0.003^d^
C18:2n6t	1.7336 ± 0.0771^b^	1.3529 ± 0.0485^c^	1.9892 ± 0.057^a^	1.1826 ± 0.0251^d^	1.0381 ± 0.059^e^	1.1613 ± 0.0273^d^	0.9931 ± 0.0676^e^	0.9975 ± 0.0509^e^
C18:2n6c	4.0985 ± 0.107^c^	4.8013 ± 0.1625^a^	5.0663 ± 0.1348^a^	4.1301 ± 0.0644^c^	3.946 ± 0.2245^cd^	4.4234 ± 0.1041^b^	3.7828 ± 0.2577^d^	3.7999 ± 0.194^d^
C18:3n6	0.1697 ± 0.0076^c^	0.2306 ± 0.0078^b^	0.2598 ± 0.0074^a^	0.1069 ± 0.0024^d^	0.0879 ± 0.005^e^	0.0416 ± 0.001^f^	0.0356 ± 0.0025^f^	0.0358 ± 0.0018^f^
C18:3n3	0.3259 ± 0.0145^e^	0.6718 ± 0.0241^cd^	0.7171 ± 0.0191^c^	0.7987 ± 0.0128^b^	0.6243 ± 0.0355^d^	0.9243 ± 0.0218^a^	0.7904 ± 0.0539^b^	0.7894 ± 0.0406^b^
C20:2	0.635 ± 0.0166^d^	1.0435 ± 0.0353^a^	0.9861 ± 0.0282^a^	0.6534 ± 0.0065^d^	0.7131 ± 0.0406^c^	1.0165 ± 0.0239^a^	0.8693 ± 0.0592^b^	0.8732 ± 0.0446^b^
C20:3n6	0.1924 ± 0.0086^b^	0.2457 ± 0.0083^a^	0.2543 ± 0.0068^a^	0.1678 ± 0.0036^c^	0.1264 ± 0.0072^d^	0.1853 ± 0.0044^b^	0.1585 ± 0.0108^c^	0.1593 ± 0.0081^c^
C20:4n6	0.3417 ± 0.0152^e^	0.6835 ± 0.0245^ab^	0.5255 ± 0.0151^d^	0.6207 ± 0.0197^c^	0.6675 ± 0.038^b^	0.7224 ± 0.017^a^	0.6178 ± 0.0421^c^	0.6206 ± 0.0317^c^
C20:3n3	0.026 ± 0.0007^f^	0.1056 ± 0.0036^b^	0.1322 ± 0.0036^a^	0.0655 ± 0.0014^c^	0.0699 ± 0.004^c^	0.0586 ± 0.0014^d^	0.0501 ± 0.0034^e^	0.0504 ± 0.0026^e^
C22:2	0.2014 ± 0.0053^b^	0.0484 ± 0.0018^c^	0.3642 ± 0.0097^a^	0.0251 ± 0.0006^e^	0.0066 ± 0.0004^f^	0.0343 ± 0.0008^d^	0.0293 ± 0.002^de^	0.0291 ± 0.0015^e^
SFA	42.1041 ± 1.4782^a^	40.9931 ± 1.0017^a^	40.9428 ± 0.6131^a^	36.2735 ± 0.513^b^	35.3486 ± 2.011^b^	37.5061 ± 0.8825^b^	32.0742 ± 2.1854^c^	32.0188 ± 1.6446^c^
MUFA	45.4127 ± 1.1295^bc^	44.7223 ± 1.3671^cd^	45.0068 ± 1.0022^bcd^	48.2764 ± 1.0116^ab^	47.8634 ± 2.723^abc^	48.8706 ± 1.1499^a^	41.7928 ± 2.8475^d^	41.7812 ± 2.143^d^
PUFA	7.7628 ± 0.0273^d^	9.2201 ± 0.1192^b^	10.3376 ± 0.0655^a^	7.7766 ± 0.1209^d^	7.3027 ± 0.4155^d^	8.59 ± 0.2021^c^	7.3459 ± 0.5005^d^	7.3291 ± 0.3767^d^

Note

The values denote mean ± standard deviation, *n* = 3. Means within a same row with alphabets (a, b, c) are significantly different (*p* < .05).

Abbreviations: MUFA, monounsaturated fatty acid; PUFA, polyunsaturated fatty acids; SFA, saturated fatty acids.

The SFA seen in the samples with the highest content was palmitic acid (C16:0). The content of monounsaturated fatty acid (MUFA) in the samples timed 0, 5, 10, 15, 20, 25, 30 and 35 min was 45.41%, 44.72%, 45.01%, 48.28%, 47.86%, 48.87%, 41.79%, and 41.78%, respectively. Elaidic acid (C18:1n9c) was the most abundant MUFA in stir‐frying Tan sheep meat. The contents of polyunsaturated fatty acids (PUFA) in the samples timed 0, 5, 10, 15, 20, 25, 30, and 35 min were 7.76%, 9.22%, 10.34%, 7.78%, 7.30%, 8.59%, 7.35%, and 7.33%, respectively. The most PUFA seen in stir‐frying Tan sheep meat was linoleic acid (C18:2n6c), and the least was docosahexaenoic acid (C22:6n3).

#### 3.1.3 Amino acid analysis of mutton sao zi

Cooking causes protein denaturation and degradation in meat, releasing some free amino acids to participate in flavor formation (Zhao et al., 2017). Amino acids are nutrient components of mutton and precursors for aroma compounds. They directly contribute to the flavor of meat and can be used to evaluate the quality of meat products (Khan et al., 2015). The composition of 18 amino acids in stir‐fried mutton sao zi is presented in Table 3. The data showed that amino acid content in mutton increased significantly (*p* < .05) after stir‐frying, due to moisture loss (Table 1). Stir‐frying caused a significantly lower moisture content, as expected, from 69.79% for stir‐frying 0 min samples to 47.11% for stir‐frying 35 min samples, and consequently a significantly higher amino acids content. However, moisture and amino acid contents showed no significant differences (*p* > .05) between stir‐frying 30 min and 35min samples. For the amino acids composition as stir‐frying continued, the most in mutton sao zi were ASP, GLU, LYS, and ARG (>2 g/100 g). A similar trend was observed in pork, where ASP and GLU were present at a higher level (Purchas et al., 2009). Glutamine is the most abundant amino acid in the body of mammals, comprising nearly 60% of the free intracellular amino acids in skeletal muscle (Lopes et al., 2015). In contrast, HIS, Trp, and Cys were recorded at the lowest contents (<1 g/100 g), which might be due to that Trp and Cys were destroyed by thermal hydrolysis (Moughan, 2003). However, in this study, cysteine was completely lost when the muscle tissue was hydrolyzed before any acid oxidation. Most of the analyzed 18 amino acids changed significantly as a result of stir‐frying, namely, stir‐frying can change the content of amino acids in mutton sao zi. Over the past 15 years, increasing evidence suggests that 6 out of the 15 amino acids measured in samples of beef changed significantly with cooking (Lopes et al., 2015).

**Table 3:** The 18 amino acid composition (g/100g of total amino acids) in mutton sao zi from different processing time (means, ±*SD*)

	Stir‐frying 0min	Stir‐frying 5min	Stir‐frying 10min	Stir‐frying 15min	Stir‐frying 20min	Stir‐frying 25min	Stir‐frying 30min	Stir‐frying 35min
ASP	1.8067 ± 0.0802^e^	2.4033 ± 0.1358^d^	2.8112 ± 0.0854^c^	2.8567 ± 0.0851^c^	2.9333 ± 0.1026^bc^	3.1267 ± 0.1250^a^	3.0633 ± 0.1102^ab^	3.0667 ± 0.0751^ab^
THR	0.8967 ± 0.0404^d^	1.2033 ± 0.0702^c^	1.3433 ± 0.0451^b^	1.3767 ± 0.0451^b^	1.4033 ± 0.0473^b^	1.5705 ± 0.0600^a^	1.5367 ± 0.0551^a^	1.5367 ± 0.0351^a^
SER	0.7033 ± 0.0351^d^	0.9367 ± 0.0551^c^	1.0833 ± 0.0351^b^	1.1267 ± 0.0351^b^	1.1367 ± 0.0416^b^	1.2900 ± 0.050^a^	1.2333 ± 0.0404^a^	1.2333 ± 0.0306^a^
GLU	3.0767 ± 0.1358^d^	4.2543 ± 0.2364^c^	4.7933 ± 0.1504^b^	4.9033 ± 0.1501^b^	4.9317 ± 0.1803^b^	5.3333 ± 0.215^a^	5.31 ± 0.1908^a^	5.3133 ± 0.1201^a^
GLY	0.7833 ± 0.0351^e^	1.0533 ± 0.0603^d^	1.1733 ± 0.0351^c^	1.2702 ± 0.0401^b^	1.2833 ± 0.0473^b^	1.6267 ± 0.0651^a^	1.5667 ± 0.0551^a^	1.5667 ± 0.0351^a^
ALA	1.1067 ± 0.0503^d^	1.4821 ± 0.0854^c^	1.7167 ± 0.0503^b^	1.7567 ± 0.0551^b^	1.7633 ± 0.0666^b^	1.9801 ± 0.0803^a^	1.95 ± 0.0656^a^	1.9511 ± 0.0502^a^
VAL	0.9067 ± 0.0404^e^	1.1905 ± 0.0656^d^	1.4133 ± 0.0451^c^	1.4267 ± 0.0451^c^	1.4567 ± 0.0513^bc^	1.5804 ± 0.0608^ab^	1.5367 ± 0.0551^a^	1.5367 ± 0.0351^ab^
MET	0.5122 ± 0.0237^e^	0.6933 ± 0.0404^d^	0.8039 ± 0.0252^b^	0.7633 ± 0.0252b^c^	0.7504 ± 0.0265^c^	0.8103 ± 0.0312^b^	0.8833 ± 0.0306^a^	0.8867 ± 0.0252^a^
ILE	0.8333 ± 0.0351^e^	1.1104 ± 0.0656^d^	1.2867 ± 0.0404^c^	1.2821 ± 0.0404^c^	1.3267 ± 0.0513b^c^	1.4633 ± 0.0551^a^	1.3933 ± 0.0503^ab^	1.3967 ± 0.0351^ab^
LEU	1.6407 ± 0.0755^e^	2.1833 ± 0.1258^d^	2.5867 ± 0.0802^c^	2.5923 ± 0.0821^c^	2.7167 ± 0.0971^bc^	2.9033 ± 0.115^a^	2.7933 ± 0.1002^ab^	2.7967 ± 0.0651^ab^
TYR	0.6467 ± 0.0306^e^	0.8833 ± 0.0503^d^	1.2067 ± 0.0404^a^	1.2514 ± 0.0420^a^	0.9606 ± 0.0361^c^	1.1167 ± 0.0451^b^	1.0967 ± 0.0351^b^	1.0967 ± 0.0252^b^
PHE	0.6633 ± 0.0252^d^	0.9233 ± 0.0503^c^	1.1633 ± 0.0351^b^	1.1667 ± 0.0351^b^	1.2167 ± 0.0416^ab^	1.2513 ± 0.0515^a^	1.1633 ± 0.0404^b^	1.1667 ± 0.0252^b^
LYS	1.6733 ± 0.0751^d^	2.2433 ± 0.1258^c^	2.5626 ± 0.0854^b^	2.6003 ± 0.0802^b^	2.7103 ± 0.0985^ab^	2.8767 ± 0.115^a^	2.8233 ± 0.1002^a^	2.8267 ± 0.0651^a^
HIS	0.5833 ± 0.0252^d^	0.6833 ± 0.0404^c^	0.7403 ± 0.0202^b^	0.7716 ± 0.0205^b^	0.7903 ± 0.0265^b^	0.8531 ± 0.0305^a^	0.8600 ± 0.0322^a^	0.8601 ± 0.0203^a^
ARG	1.1267 ± 0.0503^f^	1.5337 ± 0.0854^e^	1.8267 ± 0.0603^d^	1.8742 ± 0.0631^cd^	1.9567 ± 0.0681^bc^	2.1833 ± 0.0851^a^	2.0333 ± 0.0702^b^	2.0305 ± 0.0532^b^
PRO	0.6333 ± 0.0252^f^	0.8833 ± 0.0503^e^	0.9667 ± 0.0306^d^	1.0233 ± 0.0341^cd^	1.0608 ± 0.0361^c^	1.3267 ± 0.0551^a^	1.2333 ± 0.0404^b^	1.2333 ± 0.0306^b^
Trp	0.1702 ± 0.0101^e^	0.2533 ± 0.0153^d^	0.2602 ± 0.01209^d^	0.2931 ± 0.01091^c^	0.7519 ± 0.0265^a^	0.3029 ± 0.0118^c^	0.3367 ± 0.0153^b^	0.3233 ± 0.0058^b^
Cys	0.2002 ± 0.0103^e^	0.2367 ± 0.0153^d^	0.2822 ± 0.0114^abc^	0.2617 ± 0.0135^c^	0.2724 ± 0.0135^bc^	0.3007 ± 0.0103^a^	0.2904 ± 0.0102^ab^	0.2915 ± 0.0108^ab^

Note

The values denote mean ± standard deviation, *n* = 3. Means within a same row with alphabets (a, b, c) are significantly different (*p* < .05).

### 3.2 Electronic nose response

#### 3.2.1 Response signals of E‐nose for stir‐frying mutton sao zi samples

**Figure 1:**
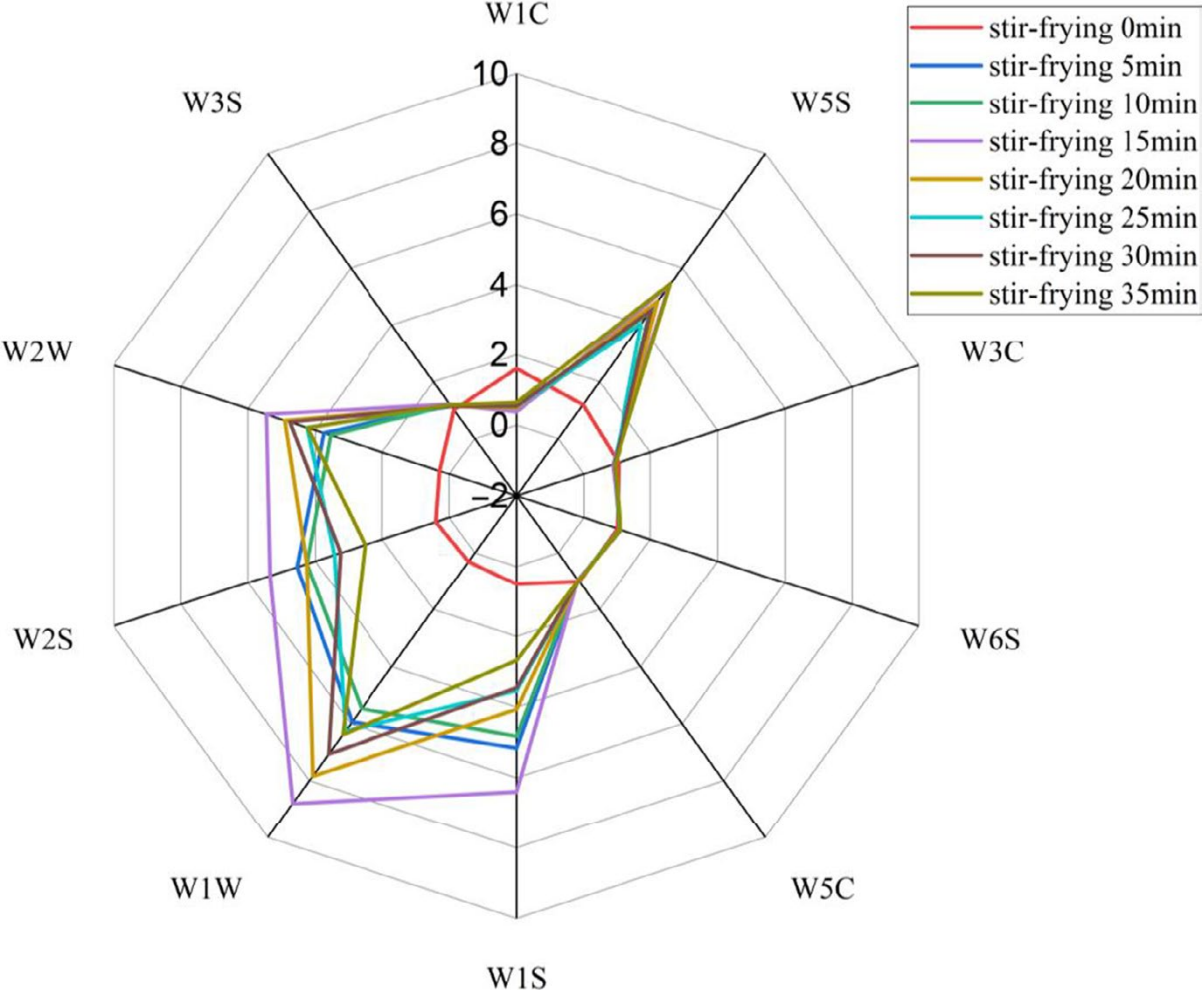
Radar plot of the response of different types of volatiles for stir‐frying mutton sao zi sample

Electronic noses are devices able to characterize and differentiate the aroma profiles of various food, especially meat and meat products. As seen in Figure 1, the response signals of E‐nose for stir‐frying mutton sao zi sample at each processing stage and the odor contour curves were different. Stir‐frying processing lowered the signal response values of W1C for 0 min sample, suggesting that stir‐frying processing could reduce aromatics as toluene (Appendix S1: Table S1). The response values of W5S, W1S, W1W, W2S, and W2W were higher than those of 0 min sample, indicating that a large number of volatile compounds, the main substances for flavor development of mutton sao zi, were produced during the stir‐frying. The response values of W5S at different stir‐frying time points higher than that at 0 min evidenced that stir‐frying method could promote the release of alcohols from mutton. Most of the alcohols were generated from lipolysis and lipid oxidation, before forming various flavor substances through the Maillard and other reactions. Lipid substances are the predecessors of flavor compounds (Silva et al., 2018; Sun et al., 2010). It could be speculated that heating is the main process producing the aroma of mutton. E‐nose showed good capacity in identifying mutton sao zi at different stir‐frying time points mainly through W1S, W1W, W2S, and W2W sensors (Figure 1). W1S, W1W, W2S, and W2W sensors gave stronger and different responses to aroma compounds of stir‐fried samples, indicating that the industrial stir‐frying mutton sao zi may have higher abundances of sulfides, terpenes, alcohols, and aromatic compounds. Meanwhile, W3C, W3S, and W5C sensors showed lower signal intensities to the samples, and no significant difference in signal intensities among samples. It was difficult to identify samples at different processing time points of industrial stir‐frying mutton sao zi by just observing the sensor signals. Hence, PCA was further used in the study.

#### 3.2.2 PCA of E‐nose data

**Figure 2:**
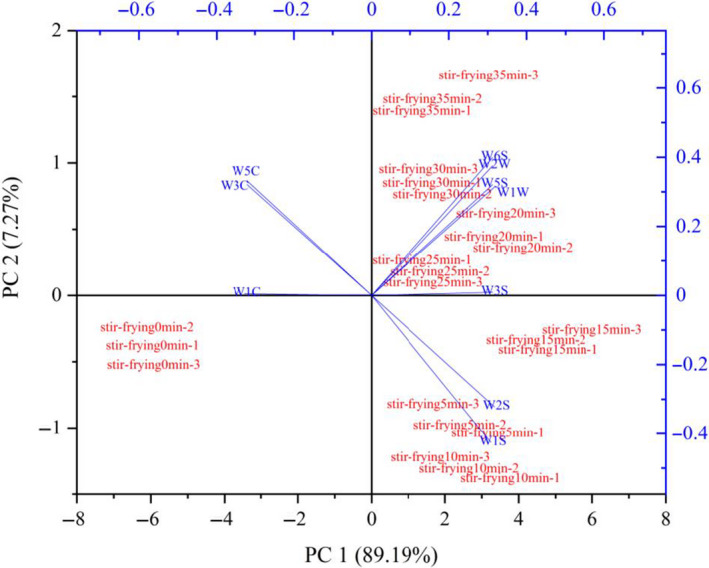
Biplot loadings and scores (PCA) of E‐nose for different processes of stir‐frying mutton sao zi. The number after the text represents the different samples with the same processing

As shown in Figure 2, the accumulative variance contribution rate of the first two principal components is > 90%, indicated that the two principal components could reflect all the characteristics of volatile odor of industrial mutton sao zi at different stir‐frying stages. The PCA showed that the two principal components were accountable for approximately 96.46% of the variability, PC1 for 89.19% and PC2 for 7.27% (Figure 2). The samples differed mainly in PC1. The data points of the samples at different processing time points were scattered, and samples of mutton sao zi at different processing stages had their own aroma regions. Samples of mutton sao zi at different stir‐frying time points (0–35 min) could be easily divided into eight groups. When the samples overlap or close to each other, it means they have similar flavor. The stir‐frying 5–35 min samples had different distribution regions from 0 min samples. W1C, W3C, and W5C were associated with the stir‐frying 0 min sample in the biplot chart, while W2S, W3S, W1S, W6S, W5S, W2W, and W1W were associated with stir‐frying 5–35 min samples. According to the analysis of E‐nose, different processing stages had significant effects on nitrogen oxides, aromatic compounds, and sulfur components of alkanes and alcohols in mutton sao zi, but little effect on alkanes, hydrides, and ammonia compounds. Thus, E‐nose is an effective tool to discriminate aroma attributes in stir‐frying mutton sao zi at different processing time points. However, it is difficult to know what the specific compounds are for these samples.

### 3.3 Volatile compounds analysis stir‐frying mutton sao zi

**Figure 3:**
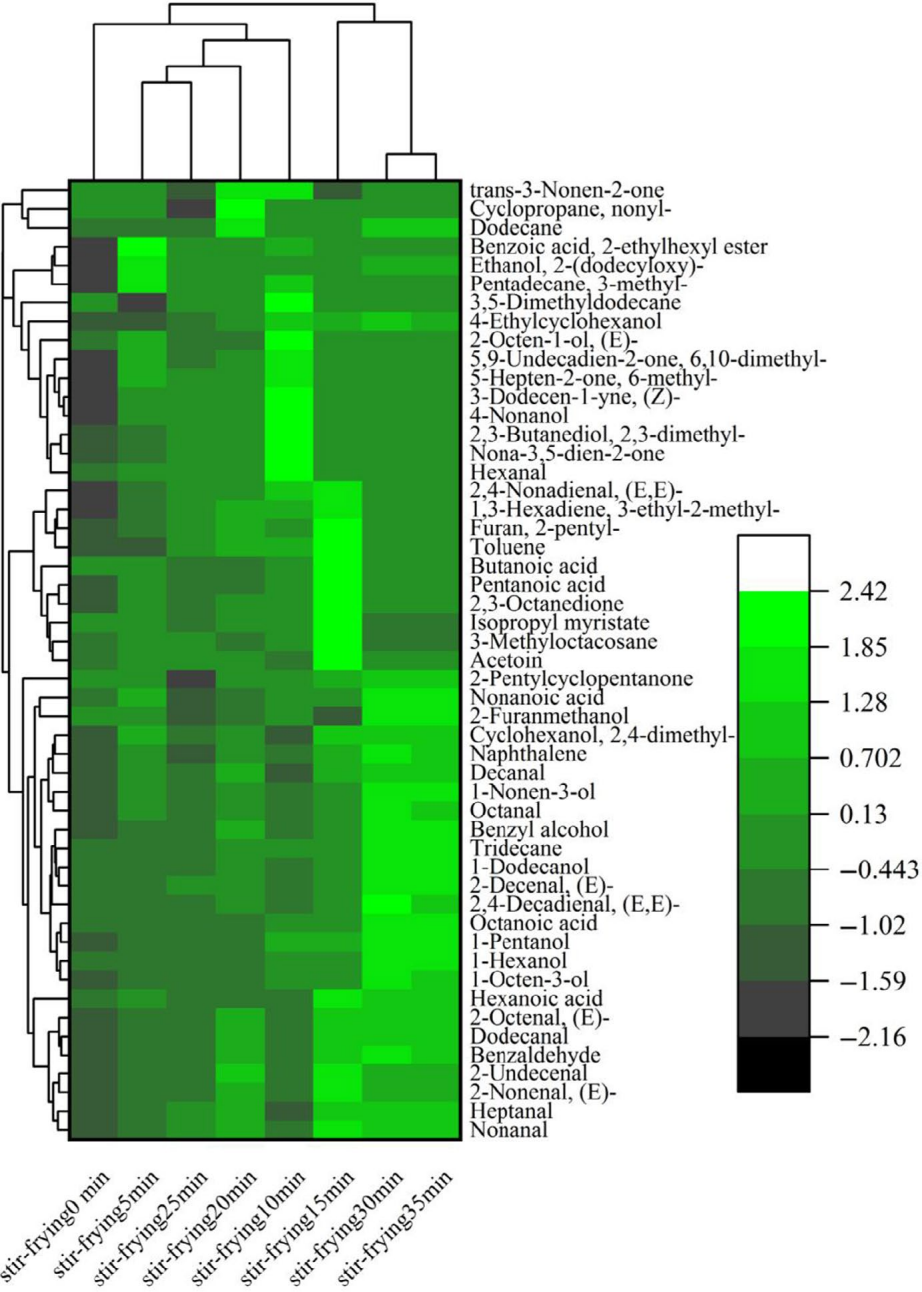
Clustering heat map of the concentration of volatile compounds in stir‐frying mutton sao zi at each processing time

**Figure 4:**
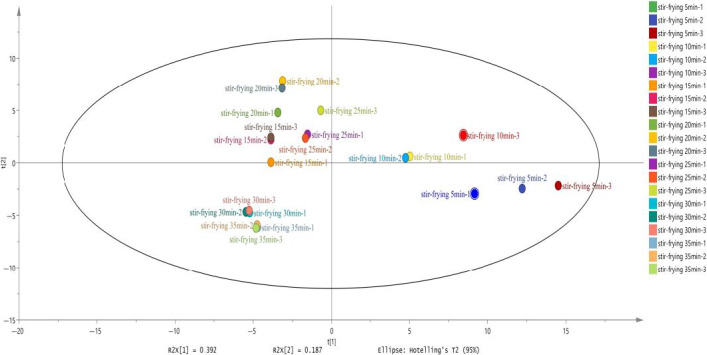
Principal component analysis of volatile compounds (GC‐MS data) of stir‐frying mutton sao zi

As we all know, the changes in flavor and surface color of meat are due to the Maillard reaction and thermal degradation of lipid, as well as the interaction between the two reaction pathways (Mottram, 1998). To have a better understanding of the flavor of the mutton sao zi, GC‐MS was utilized to analyze the volatile compounds at different stir‐frying time points. The results showed that 105 volatile compounds were identified (Appendix S1: Table S1), including 25 alcohols, 19 aldehydes, 9 acids, 10 esters, 16 ketones, 12 hydrocarbons, 8 aromatic compounds, and 6 other compounds, and 51 major volatile compounds selected by *t* test (*p* < .01) are shown in the clustering heat map (Figure 3). PCA result of GC‐MS data indicated that mutton sao zi at different stir‐frying time points separated well in terms of volatile compounds (Figure 4). Only stir‐frying 30 min and 35 min samples did not separate.

For their low perception thresholds, aldehydes often presented samples with special aroma even at trace amounts (Cai et al., 2016). The levels of aldehydes were slightly higher in the stir‐frying 15 min sample (about 70°C), possibly due to formation of these compounds with the rising temperature (Dermiki et al., 2013). Most aldehydes were derived from lipid oxidation: hexanal, nonanal, octanal, heptanal, pentanal, 2‐hexenal, and benzaldehyde (Rasinska et al., 2019), which suggested that the most aldehydes in industrial stir‐frying mutton sao zi were produced by lipid oxidation. These compounds also detected in chicken (Man et al., 2018), beef (Song et al., 2011), and pork of black‐pig (Zhao et al., 2017) have been reported in the scientific literature. It is interesting to note that the mutton sao zi samples stir‐fried in the last 10 min presented the highest amounts of hexanal, which could be the main reason for the high flavor quality. As the main aldehyde produced during lipid oxidation of meat (Shahidi & Zhong, 2010), hexanal increased, maybe attributable to lipid oxidation of unsaturated fatty acids at high temperature (30% fat in the sample). Benzaldehyde (cherry‐like odor), the one supposed to provide mutton sao zi special overall aroma, was originated from the Strecker degradation of phenylalanine. Report suggested that benzaldehyde produced almond and burnt sugar special flavor during processing (Cai et al., 2016; Pham et al., 2008). The results for volatile aldehydes from this study reveal that heating promotes rapid oxidation of polyunsaturated fatty acids, which in turn produces more free radicals capable of attacking other fatty acids less prone to oxidize, for example oleic acid, promoting formation of heptanal, octanal, and nonanal among other aldehydes (Roldan et al., 2014).

Statistical analysis showed that alcohol content of mutton sao zi was affected by thermal treatment (Appendix S1: Table S1). As shown in Figures 3 1‐octen‐3‐ol and 1‐pentanol from decomposition of lipids were detected in all the samples, while 4‐nonanol and 2,3‐dimethyl‐2,3‐butanediol were only observed in the 0–10 min sample stir‐fried at a low temperature stage (Appendix S1: Table S1 and Figure S1). Considering higher threshold value of alcohols, short straight chain alcohols might not contribute any flavor to the product. However, long straight chain alcohols like 1‐pentanol, 1‐hexanol, 1‐dodecanol, and 4‐nonanol may contribute aroma to the miso products as reported with relatively lower threshold value (Giri et al., 2010). In addition, branched‐chain alcohols like 1‐octen‐3‐ol, (E)‐2‐octen‐1‐ol, and 1‐nonen‐3‐ol might contribute significant aroma to the mutton sao zi as they're having lower threshold values. Aliphatic alcohols might contribute aroma to meat flavor through unsaturated alcohols, the threshold values of which are lower than those of saturated ones (Giri et al., 2010). For example, 1‐octen‐3‐ol contributing to a mushroom odor was detected in cooked and grilled lamb at different temperature‐time combinations in substantial amounts (Bueno et al., 2014; Wang et al., 2019). However, alcohols with higher odor thresholds, generally, are not deemed as important flavor contributors to meat products (Huang et al., 2019).

Ketones are considered to have a great impact on the aroma of meat and meat products as they give off a peculiar odor and appear in large amount in food (Man et al., 2018). 2,3‐octanedione could be counted as one of the important compounds distinguishing stir‐frying stages, because it differed significantly in content, so much that detected at some stir‐frying stages but not others (Appendix S1: Table S1). Although acetoin (sweet, buttery odor) was not a major odor influencer in grilled beef (Kilgannon et al., 2020), it presented at a high concentration in mutton sao zi and might have a subtle effect on the overall odor perception.

The esters usually have sweet and typical fruity odors generated by esterification of acids and alcohols in meat products (Domínguez et al., 2014). Only ten esters were detected by GC‐MS in mutton sao zi samples at different stir‐frying time points (Appendix S1: Table S1), including two long‐chain esters (benzoic acid, 2‐ethylhexyl ester, and Isopropyl myristate), and three lactones (delta‐nonalactone, 5‐ethyldihydro‐2(3H)‐furanone, and gamma‐dodecalactone). Among them, isopropyl myristate was at the highest level and detected in all the samples, significantly up as the temperature rose in 0–20 min and down as the temperature dropped within 25–35 min. In particular, the lactones could come from the intramolecular esterification of the hydroxy acids (Lorenzo & Domínguez, 2014).

The medium‐chain acids (C6 ~ C11) among the fatty acids are more volatile and influential to meat aroma (Song et al., 2011). In this study, butanoic acid, hexanoic acid, nonanoic acid, octanoic acid, (E,E)‐2,4‐nonadienal, and pentanoic acid medium‐chain acids were observed.

Of course, furan and pyrazine compounds, such as 2‐furanmethanol, 2(5H)‐furanone, 2‐pentyl‐furan, methyl‐pyrazine, and 2,3‐dimethyl‐pyrazine, come from Maillard reactions and Strecker degradation (Giri et al., 2010), were all detected during this research. The 2‐pentyl‐furan from linoleic acid oxidation was found attributable to fatty and meaty aroma, and its formation was normally connected with heat (Man et al., 2018). The last but not the least, some hydrocarbons were detected in mutton sao zi. Hydrocarbons showed a high proportion in the volatile compounds though, they were not considered as a major contributor to the overall flavor of samples for their high odor thresholds (Lu et al., 2008).

In this study, 3‐thiophenecarboxaldehyde was detected in late stir‐frying period, and its structure revealed that it's originated from free Cys rather than Cys‐containing peptides (Zou et al., 2019). D‐Limonene was imparted by flavorings and spice (Z. Wang et al., 2020). Volatile compounds, such as alcohols, aldehydes, ketones, acids, esters, and hydrocarbons, together were closely related to the unique flavor of mutton sao zi.

### 3.4 Correlation between E‐nose and GC‐MS

**Figure 5:**
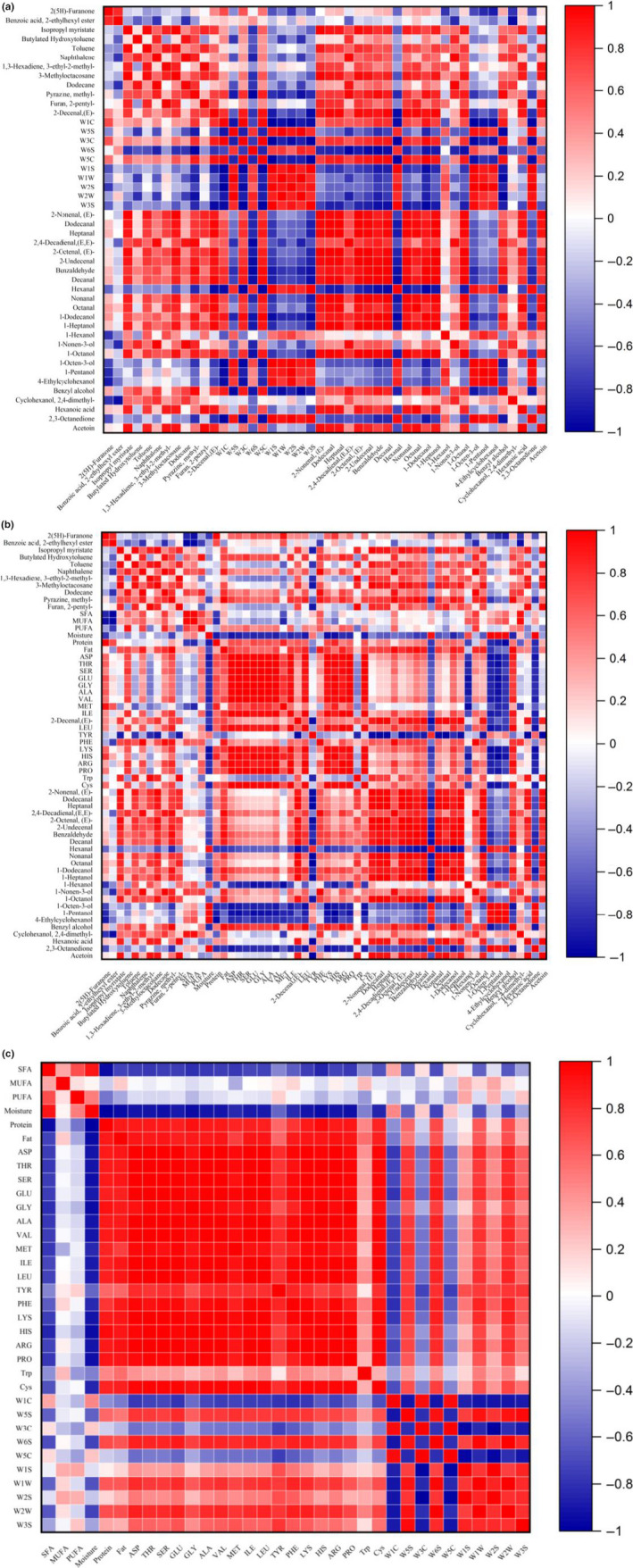
Correlation analysis among E‐nose, GC‐MS, and fatty acids, crude composition, and amino acids of stir‐frying mutton sao zi. (a) E‐nose and GC‐MS. (b) GC‐MS and fatty acids, crude composition, and amino acids. (c) E‐nose and fatty acids, crude composition, and amino acids

Based on GC‐MS analysis, highly abundant volatile components were selected to correlate with E‐nose signals, and moisture, protein, fat, fatty acids, and amino acid (Figure 5).

The highly abundant volatile components selected to correlate with E‐nose signals are shown in Figure 5a. The results indicated that the E‐nose signal intensities of W1S, W1W, W2S, W2W, and W3S sensors were in positive correlations with hexanal, 1‐hexanol, 1‐octen‐3‐ol, 1‐pentanol, 4‐ethylcyclohexanol, and 2,3‐octanedione, and negative correlations with (E)‐2‐nonenal, dodecanal, heptanal, (E,E)‐2,4‐decadienal, (E)‐2‐octenal, 2‐undecenal, benzaldehyde, decanal, nonanal, octanal, 1‐dodecanol, 1‐heptanol, 1‐nonen‐3‐ol, 1‐octanol, benzylalcohol, hexanoic acid, acetoin, (E)‐2‐decenal, 3‐methyloctacosane, dodecane, methyl‐pyrazine, naphthalene, isopropyl myristate, and butylated hydroxytoluene. In contrast, W1C, W3C, and W5C signals had negative correlation with the abundances of hexanal, 1‐hexanol, 1‐octen‐3‐ol, 1‐pentanol, 4‐ethylcyclohexanol, and 2,3‐octanedione. The results showed that W1S, W1W, W2S, W2W, and W3S sensors were sensitive to the volatile compounds at different stir‐frying time points of industrially produced mutton sao zi. This showed that E‐nose can discriminate the different stages of stir‐frying by responding specifically to volatile compounds originated from stir‐frying mutton sao zi.

### 3.5 Correlation between GC‐MS and fatty acids, crude composition, and amino acids

The interactions among volatile compounds and fat, protein, moisture, amino acid, and fatty acids had a significant influence on the flavor substances of industrial stir‐frying mutton sao zi (Figure 5b). Fat can interact with liposoluble substances, dissolve and squeeze into liposoluble substances, and therefore affect the release of liposoluble volatile compounds (Shi et al., 2020). Most compounds and fatty acids, crude composition, and amino acids had strong positive correlation with fat content, such as (E)‐2‐nonenal, heptanal, (E,E)‐2,4‐decadienal, (E)‐2‐octenal, benzaldehyde, nonanal, octanal, 1‐heptanol, 1‐nonen‐3‐ol, 1‐octanol, benzyl alcohol, hexanoic acid, 2(5H)‐furanone, isopropyl myristate, toluene, methyl‐pyrazine, 2‐pentyl‐furan, (E)‐2‐Decenal and protein, fatty acid (SFA, MUFA, and PUFA), 17 amino acids (ASP, THR, SER, GLU, GLY, ALA, VAL, MET, ILE, LEU, PHE, LYS, HIS, ARG, PRO, Trp, and Cys) shown in Figure 5b. These compounds, generally derived from the oxidation and degradation of lipid, presented grassy, fruity, and fat wax notes (Sitz et al., 2005). Fat content correlated negatively with 2‐ethylhexyl ester benzoic acid, hexanal, 1‐hexanol, 1‐octen‐3‐ol, 1‐pentanol, 4‐ethylcyclohexanol, 2,3‐octanedione, and TYR (Figure 5b).

Protein is known to bind a variety of flavor substances by reversible or irreversible binding force (Zhou et al., 2014). Protein also shows positive correlations with most compounds, amino acid and fat, except toluene, naphthalene, 3‐ethyl‐2‐methyl‐1,3‐hexadiene, dodecane, hexanal, 1‐hexanol, 1‐nonen‐3‐ol, 1‐octen‐3‐ol, 1‐Pentanol, 2,3‐octanedione, 4‐ethylcyclohexanol, 2,4‐dimethyl‐cyclohexanol, and TYR, PHE, SFA, MUFA, PUFA, and moisture (Figure 5b). The binding of nonanal to protein is the act of hydrophobic force (Ding et al., 2012), and the increase of protein concentration would inhibit the release of nonanal. Fatty acids have the ability to undergo auto‐oxidation processes which contribute to the formation of aldehydes, alcohols, ketones, and heterocyclic compounds (Shi et al., 2020). As shown in Figure 5b, SFA, MUFA, and PUFA showed strong correlation positively with dodecane, methyl‐pyrazine, butylated hydroxytoluene, naphthalene, (E,E)‐2,4‐decadienal, 2‐undecenal, benzaldehyde, hexanal, 1‐nonen‐3‐ol, 1‐octanol, 4‐ethylcyclohexanol, benzyl alcohol, 2,4‐dimethyl‐cyclohexanol, and negatively with hexanoic acid, 1‐heptanol, nonanal, octanal, dodecanal, heptanal, (E)‐2‐decenal, MET, protein, 2(5H)‐furanone, and 2‐ethylhexyl ester benzoic acid. The overall aroma of industrial mutton sao zi could be greatly influenced by these key chemical compounds through different stir‐frying stages.

### 3.6 Correlation between E‐nose and fatty acid, crude composition, and amino acids

The E‐nose analysis of the industrial stir‐frying mutton sao zi may be helpful for the release of volatile compounds from fat and proteins, and then discrimination of the overall flavor at different stir‐frying time points of mutton sao zi. The signal intensities of W1S, W1W, W2S, W2W, and W3S sensors have a positive correlation with protein, fat, and 18 amino acids (ASP, THR, SER, GLU, GLY, ALA, VAL, MET, ILE, LEU, TYR, PHE, LYS, HIS, ARG, PRO, Trp, Cys), but negative correlation with SFA and moisture (Figure 5c). It is indicated that high contents of protein, fat, SFA, and 18 amino acids contribute to E‐nose to discriminate different stir‐frying time points of mutton sao zi.

## 4 CONCLUSION

Flavor compounds of 0‐35min samples of stir‐frying mutton sao zi were determined using SPME‐GC‐MS and E‐nose. A total of 105 volatile compounds were identified by GC‐MS. Principal component analysis of GC‐MS and E‐nose data showed a good separation among groups. E‐nose discriminated the samples well because of the strong correlation of W1S, W1W, W2S, W2W, and W3S sensors with protein, fat, and 18 amino acids and some volatile compounds and the high responses to all the samples. With prolonged stir‐frying time, the protein, amino acids, and flavor compounds in mutton sao zi increased, contributing abundant unique flavors to the product. The different temperature processing the industrial stir‐frying mutton sao zi may be the critical contributor to formation of varied volatile flavor compounds at different stir‐frying time points. The correlation analysis of GC‐MS and E‐nose confirmed that E‐nose sensors were sensitive to volatile flavor compounds, which further validated the E‐nose data. Therefore, E‐nose can be used to discriminate industrial stir‐frying mutton sao zi from different processing time points. In addition, this study also emphasized the feasibility of evaluating flavor of industrial stir‐frying mutton sao zi using SPME‐GC‐MS and E‐nose.

## CONFLICTS OF INTEREST

No conflict of interest exits in the submission of this manuscript, and manuscript is approved by all authors for publication. I would like to declare on behalf of my co‐authors that the work described was original research that has not been published previously, and not under consideration for publication elsewhere. We declare that we do not have any commercial or associative interest that represents a conflict of interest in connection with the work submitted.

## Supporting information

Appendix S1
