# Supplementary material for: Characterization of flavor volatile compounds in industrial stir‐frying mutton sao zi by GC‐MS, E‐nose, and physicochemical analysis
Source: Food Sci Nutr. 2020 Dec 10;9(1):499–513. doi: 10.1002/fsn3.2019 (PMC7802549; doi:10.1002/fsn3.2019)
Supplement: Supplementary file 1 — Appendix S1 [file FSN3-9-499-s001.doc]

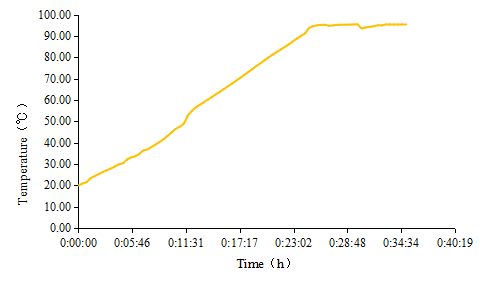


Figure S1 Variation of stir-frying temperature of industrial mutton sao zi

Table S2 Volatile compounds identified by GC–MS

| No. | Compounds name | Concentrations (µg/kg) | | | | | | | |
| --- | --- | --- | --- | --- | --- | --- | --- | --- | --- |
|  |  | stir-frying 0min | stir-frying 5min | stir-frying 10min | stir-frying 15min | stir-frying 20min | stir-frying 25min | stir-frying 30min | stir-frying 35min |
|  | **Aldehydes** | | | | | | | | |
| 1 | 2-Decenal,(E)- | 0.21±0.02^f^ | 1.39±0.19^ef^ | 5.43±1.4^d^ | 4.52±1.12^de^ | 43.65±0.28^a^ | 32.02±2.37^b^ | 34.01±0.29^b^ | 22.41±1.13^c^ |
| 2 | 2-Nonenal, (E)- | 0.71±0.07^e^ | 2.23±0.41^e^ | 7.55±0.36^d^ | 8.64±1.05^d^ | 47.25±2.76^a^ | 31.09±3.13^b^ | 21.22±2.52^c^ | 20.14±2.43^c^ |
| 3 | Dodecanal | 0.09±0.05^f^ | 0.56±0.12^f^ | 1.53±0.26^e^ | 1.44±0.17^e^ | 7.64±1.09^b^ | 5.52±0.54^c^ | 4.83±0.14^d^ | 8.37±0.45^a^ |
| 4 | Heptanal | 0.88±0.06^e^ | 5.23±0.91^e^ | 14.04±1.54^d^ | 24.36±2.56^c^ | 62.66±3.56^a^ | 48.93±3.63^b^ | 46.23±5.12^b^ | 50.36±5.32^b^ |
| 5 | 2,4-Decadienal,(E,E)- | 1.32±0.46^g^ | 6.81±0.11^f^ | 18.21±1.99^e^ | 27.99±2.81^d^ | 47.56±1.96^b^ | 60.80±6.56^a^ | 34.33±5.60^c^ | 29.47±6.98^d^ |
| 6 | 2,4-Dodecadienal, (E,E)- | - | 0.15±0.03 | - | - | - | - | - | - |
| 7 | 2,6-Octadienal, 3,7-dimethyl-, (Z)- | - | 0.15±0.04^c^ | 0.32±0.05^b^ | 0.59±0.13^a^ | - | - | - | - |
| 8 | 2-Heptenal, (Z)- | 0.60±0.14^e^ | 1.61±0.33^d^ | 4.02±0.68^c^ | 6.82±1.86^a^ | - | - | - | 6.08±1.56^b^ |
| 9 | 2-Hexenal, (E)- | - | 0.18±0.05^c^ | - | 0.60±0.08^b^ | - | 4.74±0.35^a^ | - | - |
| 10 | 2-Octenal, (E)- | 1.36±0.98^f^ | 3.03±0.65^f^ | 9.47±1.25^e^ | 13.89±0.84^d^ | 30.56±1.43^a^ | 25.55±1.22^b^ | 21.83±1.55^c^ | 23.76±2.12^bc^ |
| 11 | 2-Undecenal | 0.33±0.03^e^ | 0.79±0.17^e^ | 3.92±0.81^d^ | 2.47±0.51^de^ | 38.36±17.78^a^ | 32.47±3.37^b^ | 20.42±4.20^c^ | 18.68±2.56^c^ |
| 12 | 3-Thiophenecarboxaldehyde | - | - | - | - | - | 3.55±0.33^b^ | 1.75±0.12^c^ | 5.50±0.09^a^ |
| 13 | Benzaldehyde | 3.49±0.48^e^ | 6.38±1.71^e^ | 15.29±1.64^d^ | 22.50±2.85^d^ | 89.10±11.89^a^ | 92.73±8.98^a^ | 66.70±5.40^b^ | 36.84±4.08^c^ |
| 14 | Decanal | 0.27±0.11^g^ | 2.56±0.51^f^ | 5.28±0.19^e^ | 5.31±0.62^e^ | 22.66±2.82^a^ | 20.61±2.36^b^ | 16.5±1.42^c^ | 10.16±1.41^d^ |
| 15 | Hexanal | 15.62±1.84^e^ | 80.07±7.31^d^ | 203.32±26.01^b^ | 501.46±12.73^a^ | 194.18±12.82^b^ | 190.79±18.61^b^ | 192.27±14.44^b^ | 154.18±23.67^c^ |
| 16 | Nonanal | 6.38±0.36^g^ | 31.99±1.98^f^ | 68.45±3.90^e^ | 78.46±9.52^e^ | 337.23±13.06^a^ | 260.33±18.52^b^ | 227.62±25.15^c^ | 129.64±12.02^d^ |
| 17 | Octanal | 1.08±0.08^f^ | 5.51±1.01^f^ | 13.1±1.24^e^ | 21.15±3.96^d^ | 87.65±9.53^a^ | 51.84±4.82^c^ | 63.56±8.94^b^ | 88.22±12.74^a^ |
| 18 | Tetradecanal | - | 0.45±0.17^e^ | 3.58±0.08^c^ | - | 8.70±0.36^a^ | 6.71±1.08^b^ | 1.98±0.76^d^ | - |
| 19 | Tridecanal | - | - | - | 2.41±0.31^b^ | 7.35±0.74^a^ | 7.42±0.36^a^ | - | - |
|  | **Alcohols** | | | | | | | | |
| 20 | 1-Butanol | - | 0.35±0.07^c^ | - | 1.14±0.14^b^ | 2.51±0.26^a^ | - | - | - |
| 21 | 1-Dodecanol | 0.57±0.06^f^ | 1.45±0.61^e^ | 2.86±0.14^d^ | 1.65±0.52^e^ | 3.75±0.76^bc^ | 3.89±0.88^b^ | 3.34±1.01^cd^ | 7.17±1.76^a^ |
| 22 | 1-Heptanol | 0.57±0.02^g^ | 3.87±0.74^f^ | 7.94±1.03^e^ | 11.69±2.08^d^ | 39.3±7.08^a^ | 32.57±2.21^b^ | 27.48±3.37^c^ | 26.46±1.91^c^ |
| 23 | 1-Hexadecanol | 0.12±0.02^c^ | 0.15±0.05^c^ | - | 0.58±0.17^b^ | - | 0.68±0.17^a^ | - | - |
| 24 | 1-Hexanol | 0.91±0.10^g^ | 5.99±0.91^f^ | 14.68±1.33^cd^ | 20.9±0.99^b^ | 23.11±1.69^a^ | 16.21±4.54^c^ | 13.65±1.35^d^ | 11.2±1.34^e^ |
| 25 | 1-Nonen-3-ol | 1.22±0.08^e^ | 2.06±0.06^d^ | 2.57±0.77^d^ | 3.73±0.34^c^ | 4.66±0.07^b^ | 5.91±0.26^a^ | 3.35±0.48^c^ | 3.87±0.08^c^ |
| 26 | 1-Octanol | 1.43±0.13^g^ | 9.42±1.17^f^ | 18.41±3.71^e^ | 21.15±2.72^e^ | 66.03±5.32^a^ | 57.74±2.68^b^ | 43.97±4.43^c^ | 28.11±1.22^d^ |
| 27 | 1-Octanol, 2-butyl- | 0.17±0.04^c^ | 0.71±0.17^b^ | 0.96±0.18^a^ | 0.14±0.02^c^ | - | - | - | - |
| 28 | 1-Octen-3-ol | 15.78±1.03^f^ | 76.67±6.68^e^ | 175.93±23.32^b^ | 220.26±19.75^a^ | 159.31±18.14^b^ | 122.22±26.81^c^ | 124.67±9.98^c^ | 156.96±12.91^b^ |
| 29 | 1-Pentanol | 2.95±0.77^g^ | 17.06±2.15^f^ | 36.90±1.07^c^ | 58.96±6.49^a^ | 47.74±4.08^b^ | 31.24±3.23^d^ | 26.10±1.25^e^ | 26.91±2.92d^e^ |
| 30 | 1-Propanol, 2-methoxy- | 1.56±0.23^c^ | - | - | - | - | - | 3.63±0.08^b^ | 3.30±0.47^b^ |
| 31 | 2,3-Butanediol | - | 4.49±0.18^c^ | - | 8.29±0.59^b^ | - | 37.02±1.77^a^ | - | 38.51±3.03^a^ |
| 32 | 2,3-Butanediol, [R-(R*,R*)]- | 6.69±0.66^d^ |  | 16.39±1.34^c^ | - | 99.17±5.02^a^ | - | 64.84±6.39^b^ | - |
| 33 | 2,3-Butanediol, 2,3-dimethyl- | 0.99±0.21^d^ | 1.39±0.14^c^ | 1.66±0.26^b^ | 3.40±0.48^a^ | - | - | - | - |
| 34 | 2-Ethyl-1-hexanol | - | 1.79±0.17^c^ | 3.63±0.04^b^ | 9.12±1.96^a^ | - | - | - | - |
| 35 | 2-Furanmethanol | - | - | - | - | 7.25±1.47^d^ | 14.63±0.42^c^ | 31.78±3.56^a^ | 27.08±1.82^b^ |
| 36 | 2-Octen-1-ol, (E)- | 2.63±0.55^e^ | 13.84±2.64^cd^ | 32.24±4.57^b^ | 51.33±6.56^a^ | 16.47±2.19^c^ | 14.21±2.66^cd^ | 11.31±0.44^d^ | - |
| 37 | 4-Ethylcyclohexanol | 0.23±0.01^e^ | 0.30±0.04^e^ | 0.87±0.12^d^ | 3.92±0.67^a^ | 3.28±0.04^b^ | 3.04±0.39^b^ | 2.62±0.62^c^ | 3.31±0.11^b^ |
| 38 | 4-Nonanol | 0.75±0.06^d^ | 4.78±0.46^c^ | 9.66±1.15^b^ | 13.88±1.96^a^ | - | - | - | - |
| 39 | Benzyl alcohol | 2.67±0.27^f^ | 6.40±1.39^e^ | 10.12±2.89^d^ | 9.00±1.16^de^ | 32.02±1.09^b^ | 48.91±1.51^a^ | 31.41±2.26^b^ | 24.51±2.54^c^ |
| 40 | Cyclohexanol, 2,4-dimethyl- | 0.11±0.02^f^ | 0.63±0.02^e^ | 3.31±0.39^a^ | 1.52±0.18^c^ | 1.76±0.29^b^ | 1.88±0.41^b^ | 0.9±0.26^d^ | 1.03±0.39^d^ |
| 41 | Ethanol, 2-(dodecyloxy)- | 0.31±0.01^d^ | 0.39±0.14^c^ | 0.91±0.24^a^ | 0.39±0.14^c^ | - | - | 0.64±0.09^b^ | 0.59±0.04^b^ |
| 42 | Linalool | 0.11±0.05^b^ | - | 0.74±0.16^a^ | - | - | - | - | - |
| 43 | n-Heptadecanol-1 | - | 0.91±0.06^b^ | 1.42±0.14^a^ | - | - | - | - | - |
| 44 | trans-Linalool oxide (furanoid) | - | 1.03±0.13^c^ | 2.33±0.18^b^ | 3.82±0.25^a^ | - | - | - | - |
|  | **Acids** | | | | | | | | |
| 45 | 2,4-Nonadienal, (E,E)- | 0.31±0.05^e^ | 2.29±0.77^d^ | 5.53±1.46^c^ | 9.67±2.32^a^ | 8.73±1.36^b^ | - | - | 8.68±0.66^b^ |
| 46 | Acetic acid | 3.99±0.73^b^ | 1.73±0.58^c^ | 3.45±0.16^b^ | 3.45±0.49^b^ | 18.72±1.25^a^ | - | - | 19.65±2.79^a^ |
| 47 | Acetic acid, diethyl- | 0.99±0.09 | - | - | - | - | - | - | - |
| 48 | Butanoic acid | 1.62±0.19^d^ | - | - | - | 17.34±0.43^a^ | 8.80±0.54^c^ | 12.78±1.31^b^ | 17.24±2.51^a^ |
| 49 | Hexanoic acid | 4.94±0.23^e^ | 16.06±2.37^bc^ | 28.98±3.02^a^ | 7.20±0.33^e^ | 30.52±1.78^a^ | 14.98±2.52^c^ | 18.53±1.6^b^ | 11.17±1.34^d^ |
| 50 | Nonanoic acid | 0.77±0.13^e^ | 1.71±0.06^d^ | 2.73±0.42^b^ | - | 2.14±0.18^c^ | 1.77±0.39^d^ | 1.95±0.26^cd^ | 4.11±0.34^a^ |
| 51 | Octanoic acid | 2.08±0.33^d^ | 3.96±0.48^b^ | 6.40±1.22^a^ | - | 3.62±0.48^b^ | 3.05±0.57^c^ | 2.93±0.33^c^ | 1.29±0.43^e^ |
| 52 | Pentanoic acid | 0.40±0.24^e^ | 0.70±0.12^e^ | - | 5.96±0.59^a^ | 3.26±0.33^b^ | 1.34±0.23^d^ | 2.03±0.12^c^ | - |
| 53 | Tetradecanoic acid | - | - | 1.99±0.28^b^ | - | - | - | - | 4.69±0.01^a^ |
|  | **Ketones** | | | | | | | | |
| 54 | 2,3-Octanedione | 19.55±2.85^e^ | 91.57±5.22^d^ | 184.72±9.97^b^ | 322.94±29.37^a^ | 180.86±10.78^b^ | 114.87±15.16^c^ | 119.66±7.51^c^ | 86.63±2.41^d^ |
| 55 | 2-Decanone | - | - | - | - | 13.54±1.51^a^ | 10.89±3.86^b^ | 9.38±1.87^c^ | 8.8±0.78^c^ |
| 56 | 2-Heptadecanone | - | - | - | - | 3.62±0.78^b^ | 3.33±0.89^bc^ | 3.02±0.38^c^ | 7.05±1.13^a^ |
| 57 | 2-Pyrrolidinone | - | - | 0.11±0.01^c^ | 1.48±0.05^b^ | 3.91±0.07^a^ | - | - | - |
| 58 | 2-Pentylcyclopentanone | - | - | - | - | 5.29±0.92^a^ | 4.60±0.98^b^ | 3.78±0.50^c^ | 1.08±0.25^d^ |
| 59 | 2-Undecanone | 0.25±0.08^d^ | 0.37±0.07^d^ | - | 0.73±0.26^d^ | 15.59±1.83^b^ | 12.87±1.09^c^ | 19.88±0.99^a^ | 16.87±2.66^b^ |
| 60 | 3-Ethyl-2-pentadecanone | - | 0.32±0.07^b^ | - | - | - | - | - | 1.45±0.33^a^ |
| 61 | 3-Ethyl-2-tridecanone | - | - | - | - | - | - | - | - |
| 62 | 3-Octanone, 2-methyl- | - | 2.41±0.11^c^ | - | 10.63±1.53^a^ | - | - | - | 6.92±1.94^b^ |
| 63 | 5,9-Undecadien-2-one, 6,10-dimethyl- | 0.16±0.03^e^ | 0.53±0.21^d^ | 2.76±0.18^a^ | 2.67±0.69^a^ | - | 1.60±0.33^c^ | 2.29±0.51^b^ | - |
| 64 | 5,9-Undecadien-2-one, 6,10-dimethyl-, (Z)- | 0.27±0.17^c^ | - | - | - | 1.62±0.44^a^ | 1.24±0.43^b^ | - | - |
| 65 | 5-Hepten-2-one, 6-methyl- | 0.28±0.04^d^ | 0.49±0.09^c^ | 1.37±0.61^b^ | 2.19±0.79^a^ | - | - | - | - |
| 66 | Acetoin | 11.55±0.23^f^ | 25.77±1.49^e^ | 46.69±2.33^de^ | 50.57±6.89^d^ | 377.1±16.23^a^ | 162.02±21.73^b^ | 175.09±34.08^b^ | 136.86±25.38^c^ |
| 67 | Nona-3,5-dien-2-one | 1.11±0.09^c^ | 2.42±0.51^b^ | 2.63±0.72^b^ | 7.46±2.38^a^ | - | - | - | - |
| 68 | 2(5H)-Furanone | - | - | - | 1.22±0.27^c^ | 1.56±0.21^b^ | 1.64±0.27^b^ | 2.65±0.7^3a^ | 2.66±0.31^a^ |
| 69 | trans-3-Nonen-2-one | - | 0.58±0.05^e^ | 2.97±0.61^b^ | 4.15±0.33^a^ | 1.19±0.12^d^ | 2.98±0.10^b^ | 2.35±0.59^c^ | - |
|  | **Esters** | | | | | | | | |
| 70 | .delta.-Nonalactone | - | - | - | - | 2.52±0.03^a^ | 1.39±0.14^b^ | 1.54±0.5^b^ | - |
| 71 | .gamma.-Dodecalactone | - | - | 1.17±0.21^b^ | 0.91±0.18^c^ | - | - | 3.14±1.21^a^ | - |
| 72 | 2(3H)-Furanone, 5-ethyldihydro- | - | 0.39±0.07^e^ | 0.81±0.18^d^ | 0.58±0.23^d^ | 3.77±0.68^a^ | 3.35±1.06^b^ | 2.98±0.74^c^ | 2.87±0.36^c^ |
| 73 | Benzoic acid, 2-ethylhexyl ester | 0.15±0.03^d^ | 0.19±0.03^d^ | 0.86±0.23^a^ | 0.46±0.05^c^ | 0.48±0.01^c^ | 0.35±0.13^c^ | 0.71±0.21^b^ | - |
| 74 | Carbamodithioic acid, diethyl-, methyl ester | - | - | 1.16±0.48^b^ | 2.05±0.21^a^ | - | - | - | - |
| 75 | Carbonic acid, undecyl vinyl ester | - | 2.19±0.54^b^ | 7.40±0.16^a^ | - | - | - | - | - |
| 76 | Isopropyl myristate | 3.96±0.47^e^ | 12.43±1.79^c^ | 12.35±0.64^c^ | 8.66±1.73^d^ | 36.15±1.20^a^ | 23.45±1.42^b^ | 13.09±3.11^c^ | 6.44±1.79^d^ |
| 77 | l-Pantoyl lactone | - | - | - | - | - | - | 11.42±1.28^a^ | 6.02±0.61^b^ |
| 78 | n-Caproic acid vinyl ester | - | 2.10±0.28^c^ | 63.08±8.43^b^ | 78.45±13.1^a^ | - | - | - | - |
| 79 | Nonanoic acid, 2-oxo-, methyl ester | - | 0.90±0.29^b^ | - | 3.22±1.32^a^ | - | - | - | - |
|  | **Aromatic compounds** | | | | | | | | |
| 80 | Benzene, (3,3-dimethyldecyl)- | - | 0.09±0.03^c^ | 0.46±0.19^a^ | 0.28±0.12^b^ | - | - | - | - |
| 81 | Benzene, 1,3-dimethyl- | - | 0.96±0.14^b^ | - | - | 9.53±0.52^a^ | - | - | - |
| 82 | Butylated Hydroxytoluene | 1.34±0.06^d^ | 5.23±2.88^d^ | 22.96±2.96^b^ | 17.97±1.81^bc^ | 15.85±1.99^c^ | 107.25±11.22^a^ | 20.89±1.06^bc^ | 4.92±0.38^d^ |
| 83 | Mesitylene | - | 0.77±0.19 | - | - | - | - | - | - |
| 84 | p-Xylene | 2.41±0.33^b^ | - | - | 1.85±0.34^c^ | 5.78±0.25^a^ | - | - | - |
| 85 | Styrene | 1.43±0.18 | - | - | - | - | - | - | - |
| 86 | Toluene | 83.33±5.18^a^ | 1.5±0.23^d^ | 1.75±0.55^d^ | 7.47±0.59^c^ | 13.39±1.12^b^ | 9.83±0.31^bc^ | 6.83±1.86^c^ | - |
| 87 | Naphthalene | 1.19±0.10^f^ | 2.54±1.02^e^ | 2.80±0.39^e^ | 3.60±0.30^d^ | 5.34±1.06^c^ | 7.39±1.46^b^ | 3.28±0.66^de^ | 9.51±2.39^a^ |
|  | **Hydrocarbons** | | | | | | | | |
| 88 | 1,3-Hexadiene, 3-ethyl-2-methyl- | 0.31±0.01^d^ | 0.60±0.08^d^ | 1.63±0.24^c^ | 3.58±0.97^b^ | 5.42±1.18^a^ | 3.63±1.08^b^ | 3.65±0.29^b^ | - |
| 89 | 1-Tetradecen-3-yne |  | - | 2.35±0.76^b^ | 3.02±0.67^a^ | - | - | - | - |
| 90 | 1-Tridecene | - | - | - | 0.61±0.18^c^ | 1.70±0.22^b^ | - | - | 15.66±1.50^a^ |
| 91 | 2,6,10-Trimethyltridecane | 0.41±0.23^b^ | - | - | - | - | - | - | 42.65±4.08^a^ |
| 92 | 3,5-Dimethyldodecane | - | 1.56±0.17^c^ | 2.06±0.63^b^ | 2.59±0.97^a^ | - | - | - | - |
| 93 | 3-Dodecen-1-yne, (Z)- | - | 0.35±0.05^c^ | 0.87±0.09^b^ | 1.10±0.39^a^ | - | - | - | - |
| 94 | 3-Methyloctacosane | 35.03±3.85^cd^ | 8.87±0.20^f^ | 9.92±1.72^f^ | 21.99±2.06^e^ | 100.84±8.62^a^ | 65.53±1.19^b^ | 37.62±4.31^c^ | 29.91±1.53^d^ |
| 95 | Cyclopentane, pentyl- | 0.32±0.16 | - | - | - | - | - | - | - |
| 96 | Cyclopropane, nonyl- | - | - | - | - | 1.14±0.13^a^ | 0.94±0.40^b^ | 0.92±0.28^b^ | - |
| 97 | D-Limonene | 0.91±0.17^c^ | - | 11.93±1.61^a^ | - | - | - | - | 3.46±0.35^b^ |
| 98 | Dodecane | 1.34±0.6^e^ | 1.1±0.31^e^ | 2.77±1.22^d^ | 5.84±0.93^c^ | 9.26±1.04^b^ | 18.86±1.31^a^ | 3.85±0.07^d^ | 3.54±0.21^d^ |
| 99 | Pentadecane, 3-methyl- | 1.01±0.09^d^ | 1.26±0.17^d^ | 4.48±0.82^a^ | 2.88±1.13^b^ | - | - | 2.17±0.14^c^ | - |
|  | **Others** | | | | | | | | |
| 100 | Diallyl disulphide | 0.15±0.01 | - | - | - | - | - | - | - |
| 101 | Dodecyl heptyl ether |  | - | - | 1.69±0.07 | - | - | - | - |
| 102 | Eicosyl isopropyl ether | 0.68±0.30^a^ | - | - | - | - | - | - | 24.08±1.88^a^ |
| 103 | Pyrazine, methyl- | - | 4.15±0.36^f^ | 6.44±1.08^e^ | 12.57±1.23^d^ | 17.17±0.26^b^ | 19.32±1.87^a^ | 15.40±1.93^c^ | 11.65±2.41^d^ |
| 104 | Pyrazine, 2,3-dimethyl- | - | - | - | - | 1.52±0.37^c^ | 2.65±0.22^b^ | 1.17±0.08^d^ | 4.28±0.37^a^ |
| 105 | Furan, 2-pentyl- | 0.55±0.23^f^ | 1.64±0.23^e^ | 5.57±0.65^d^ | 8.07±2.05^c^ | 13.9±1.64^a^ | 8.58±1.43^b^ | 9.45±0.54^bc^ | - |

-: not found.
